# Prognostic significance of nm23-H1 expression in oral squamous cell carcinoma

**DOI:** 10.1038/sj.bjc.6601808

**Published:** 2004-05-04

**Authors:** Y-F Wang, K-C Chow, S-Y Chang, J-H Chiu, S-K Tai, W-Y Li, L-S Wang

**Affiliations:** 1 Department of Otolaryngology, Taipei Veterans General Hospital, National Yang-Ming University, Taipei 112, Taiwan, ROC; 2 Institute of Clinical Medicine, National Yang-Ming University, Taipei 112, Taiwan, ROC; 3 Institute of Biomedical Sciences, National Chung Hsing University, Taichung 402, Taiwan, ROC; 4 Institute of Traditional Medicine, National Yang-Ming University, Taipei 112, Taiwan, ROC; 5 Department of Pathology, Taipei Veterans General Hospital, National Yang-Ming University, Taipei 112, Taiwan, ROC; 6 Division of Chest Surgery, Department of Surgery, Taipei Veterans General Hospital and National Yang-Ming University, Taipei 112, Taiwan, ROC

**Keywords:** oral squamous cell carcinoma (OSCC), nm23, metastasis, prognosis

## Abstract

Recent studies indicated nm23-H1 played a role in cancer progression. Therefore, we investigated clinical significance of nm23-H1 expression in oral squamous cell carcinoma (OSCC). In total, 86 OSCC specimens were immunohistochemically stained with nm23-H1-specific monoclonal antibodies. Immunohistochemical staining of nm23-H1 was confirmed by immunoblotting. The relations between nm23-H1 expression and clinicopathologic variables were evaluated by *χ*^2^ analysis. As increased size of primary tumour could escalate metastatic potential and the data of patients at the late T stage might confound statistical analyses, we thus paid special attention to 54 patients at the early T stage of OSCC. Statistical difference of survival was compared by a log-rank test. Immunohistochemically, nm23-H1 expression was detected in 48.8% (42 out of 86) of tumorous specimens. It positively correlated with larger primary tumour size (*P*=0.03) and inversely with cigarette-smoking habit (*P*=0.042). In patients at the early T stage, decreased nm23 expression was associated with increased incidence of lymph node metastasis (*P*=0.004) and indicated poor survival (*P*=0.014). Tumour nm23-H1 expression is a prognostic factor for predicting better survival in OSCC patients at the early T stage, which may reflect antimetastatic potential of nm23. Therefore, modulation of nm23-H1 expression in cancer cells can provide a novel possibility of improving therapeutic strategy at this stage. In addition, our results further indicated cigarette smoking could aggravate the extent of nm23-H1 expression and possibly disease progression of OSCC patients.

Oral squamous cell carcinoma (OSCC) is the most common malignant tumour of the oral cavity. The incidence of OSCC differs among the various regions of the world and the relative frequency ranges from less than 0.1% to over 40% (Pindborg, 1977; Sankaranarayanan, 1990). In Taiwan, OSCC constitutes a significant portion of all malignancies (Tsay and Chiu, 1990). Since 1991, this disease has been the fifth leading cause of cancer death in males, and the seventh in the whole population (Hsieh *et al*, 2001).

Despite the curative surgical resection and multitherapeutic modalities, the overall survival has not improved substantially in the last two decades (Zakrzewska, 1999). The major causes of treatment failure are early lymphatic involvement and distant metastasis. It has been reported that more than half of the patients with resectable OSCC had locoregional spreading at diagnosis (Chen *et al*, 1999; Wang *et al*, 2002). Moreover, the differential responses between patients with a small oral tumour but evident metastasis having poorer prognosis and those with a large primary lesion but without detectable spreading obtaining better survival suggest a basic mechanism underlying this difference. A biomarker that can reflect this mechanism, forecast the status of disease progression and the potential of cancer metastasis would be imperative to commence the optimal treatment and improve survival significantly.

The nm23 gene was originally identified by differentiating cDNA libraries from strongly and weakly metastatic murine melanoma cell lines (Steeg *et al*, 1988a). Higher expression of nm23 was found in weakly metastatic cells. Further studies confirmed the antimetastatic effect in animal models (Steeg *et al*, 1988b; Caligo *et al*, 1992) and in human carcinomas (Bevilacqua *et al*, 1989; Caligo *et al*, 1997). The consequent protein analysis showed that nm23 not only had the sequence homologous to nucleoside diphosphate kinase (NDPK) but also had NDPK activity to convert nucleoside diphosphates to nucleoside triphosphates with an expense of ATP (Gilles *et al*, 1991; Cipollini *et al*, 1997). In human, nm23/NDPK family consists of at least eight homologues (nm23-H1 to nm23-H8) (Lacombe *et al*, 2000). Among these homologues, nm23-H1 and nm23-H2, sharing an 88% identity with each other, are further shown closely associated with antimetastatic potential (Stahl *et al*, 1991). Nonetheless, clinical studies of nm23 expression in various cancers did not fully support such notion. A few clinical results indeed indicated that nm23 expression, as anticipated from the *in vitro* investigations, correlated with better prognosis (Bevilacqua *et al*, 1989; Barnes *et al*, 1991; Hirayama *et al*, 1991; Nakayama *et al*, 1992; Campo *et al*, 1994; Hsu *et al*, 1999). Results from other studies, on the contrary, suggested that nm23 might facilitate tumour development and disease progression (Hailat *et al*, 1991; Zou *et al*, 1993; Müller *et al*, 1998; Pavelic *et al*, 2000). The discrepancy may be due to, in part, the difference of study design and, in part, study end point selected. It is worth noting that tumour development is a multifactorial process, and it is likely that a specific factor might be able to play a certain role only within a defined period of disease progression. At other time, the nature of this specific factor might change.

Expression of nm23 and its clincopathologic significance have not been well established in patients with OSCC. Therefore, in this study, we used immunohistochemical (IHC) method to examine the nm23-H1 expression in surgical specimens of OSCC patients. Expression of nm23-H1 was further confirmed by immunoblotting. Correlations between nm23-H1 and respective clinicopathologic parameters as well as prognostic significance of nm23-H1 in OSCC patients were assessed by statistical analysis, in which nm23 expression was used as the determinant to categorise the functional groups. The biological role of nm23-H1 in OSCC was then deduced following these analyses.

## MATERIALS AND METHODS

### Patients and tissue specimens

From October 1984 to June 1998, clinicopathologic data and tissue specimens from 86 consecutive patients diagnosed as OSCC were collected. All patients were pathologically confirmed OSCC. The preoperative workup consisted of physical examination, intraoral biopsy, computed tomography (CT) scan of head and neck, sonography of the abdomen, chest radiography and radioisotopic bone scan of whole body. All patients underwent surgical resection of tumours. Comprehensive or selective neck dissection was performed for suspected cervical lymphadenopathy or prophylactic neck management (Scully and Porter, 2000). Local irradiation and/or systemic cisplatin-based chemotherapy were postoperatively administered for patients with advanced stages of the disease, insufficient margins (<2 cm) of curative resection or tumour recurrence. Nevertheless, no patient received neoadjuvant therapy in this cohort. After treatment, all patients were followed routinely. Stages of the disease were categorised based on TNM staging system (Fleming *et al*, 1997). According to Broders' criteria (Anneroth *et al*, 1987), differentiation pattern of the malignancy was classified into three histologic grades: well, moderately and poorly differentiated squamous cell carcinoma. Of 86 surgical specimens, 50 were well and 36 were moderately differentiated carcinomas. No poorly differentiated OSCC was detected. Tumour recurrence and metastasis were identified when physical examination, endoscopy, chest radiography, CT scan of head and neck, sonography of the abdomen or radioisotopic bone scan of whole body showed an evidence. If possible, biopsy confirmation was performed. Medical Ethical Committee has approved this protocol and the written informed consent has been obtained from every patient before surgery. A single-blinded procedure was followed for immunohistochemical staining, immunoblotting and statistical analysis.

### Immunohistochemical staining

Expression of nm23-H1 in the pathologic sections was detected by an immunoperoxidase method as previously described (Wang *et al*, 1999a, 2002). Paraffin blocks were sectioned at the thickness of 4 *μ*m. The wax was melted at 65°C overnight. The sections were deparaffinised in xylene, and xylene was subsequently removed with absolute ethanol. The slides were then incubated with mouse monoclonal antibodies specific to nm23-H1 (Santa Cruz Biotechnology, CA, USA) and followed by biotin-conjugated goat anti-mouse immunoglobulin and horseradish peroxidase (HRP)-conjugated streptavidin (DAKO, Glostrup, Denmark). Aminoethylcarbazole was used as chromogenic substrate and red precipitate was identified as positive staining. The specimens were counterstained with haematoxylin and mounted with glycerol gelatin. In each experiment, a section of human breast cancer known to overexpress nm23-H1 was served as a positive control (DAKO, Glostrup, Denmark) and a section without adding the primary antibody was used as a negative control. Each batch of IHC contained the slides of a positive and a negative control to ensure the staining quality.

### Slide evaluation of immunohistochemical staining

**Figure 1:**
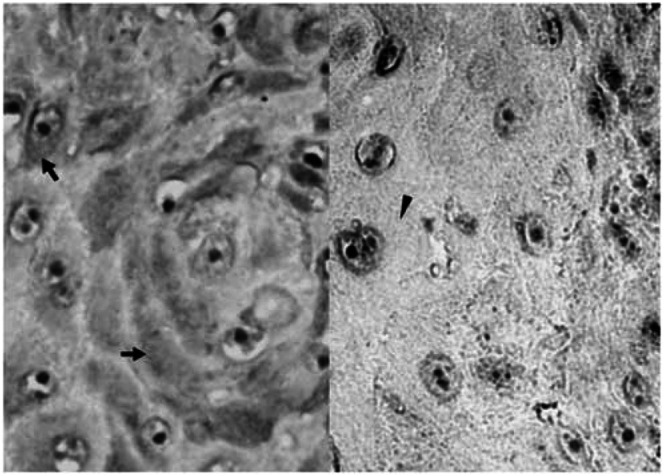
Immunohistochemical staining for nm23-H1 protein in OSCC. Left: Representative example of positive nm23-H1 expression, which demonstrated intense nm23-H1 immunoreactivity (arrows) in the cytoplasma of tumour cells. Right: Representative example of negative nm23-H1 expression. Arrowhead indicates almost no precipitate in the cytoplasma of tumour cells (original magnification × 400).

Slide evaluation has been described previously (Barnes *et al*, 1991; Hsu *et al*, 1999). Briefly, histologically nontumourous epithelium of oral tissue was served as internal negative control in each case. Under the low-power field, each slide was evaluated randomly at 10 different areas containing tumour cells by two independent investigators blinded to the clinicopathologic data. At least 100 tumour cells were examined per field. Two scoring systems, staining intensity and percentage of stained cells, were included in our study (Barnes *et al*, 1991; Moskaluk, 2002). The staining intensity was scored on a semiquantitative four-point scale as follows: 0, equivalent to the negative control; 1, weak cytoplasmic stain slightly darker than the negative control; 2, moderate stain defined as the intensity between score 1 and 3; 3, intense stain equivalent to or darker than the positive control. We use the photomicrographs of the respective four scales (0–3) as standard comparators while interpreting the slides. When there were more than 25% of cancer cells with staining intensity scored above 2–3 (Figure 1

), nm23-H1 expression was recorded positive for this patient (Wang *et al*, 1999b).

### Immunoblotting

IHC staining of nm23-H1 was then confirmed by immunoblotting in part of surgical specimens. Briefly, tissues were washed in phosphate-buffered saline and lysed in loading buffer containing 50 mM Tris (pH 6.8), 150 mM NaCl, 1 mM disodium EDTA, 5% *β*-mercaptoethanol, 1 mM phenylmethylsulphonylfluoride, 0.01% bromophenol blue, 10% glycerol and 1% SDS supplemented with leupeptin (10 *μ*g ml^−1^), aprotinin (10 *μ*g ml^−1^) and trypsin inhibitor (10 *μ*g ml^−1^). Electrophoresis was carried out in 12% polyacrylamide gel with 4.5% stacking gel. After electrophoresis, proteins were transferred to a nitrocellulose membrane. The membrane was then probed with mouse monoclonal antibodies specific to nm23-H1 (1 : 500), and then HRP-conjugated goat anti-mouse IgG (ICN Pharmaceuticals, OH, USA) (1 : 5000). The protein bands were visualised by exposing a X-Omat film (Eastman Kodak, NY, USA) with enhanced chemiluminescent reagent (PIERCE, IL, USA) to the membrane.

### Statistical analysis

The relationships between nm23-H1 expression and each of clinicopathologic parameters, including histologic grade of malignancy, primary tumour size, lymph node involvement, distant metastasis, tumour recurrence, habits of betel nut-chewing and cigarette-smoking, were analysed by *χ*^2^ with Yates' correction or Fisher's exact test (when the expected number of any cell was fewer than five). Survival curves were plotted with the method of Kaplan–Meier (Kaplan and Meier, 1958). Statistical difference of survival between different groups was compared by a log-rank test (Peto and Pike, 1973). The joint effect of clinicopathologic factors was further tested in multivariate analysis using a Cox regression model (Peto and Pike, 1973). Statistical analysis was performed by using SPSS software (version 10.0, SPSS Inc., IL, USA). Statistical significance was set at *P*-value <0.05.

## RESULTS

### Clinicopathologic characteristics

The mean age of this study group was 49.7 years (range, 24–85 years) and male–female ratio was 5.6 : 1 (73 men and 13 women). The median follow-up was 35.5 months, ranging from 1 to 165 months. Up to the time of final statistical analysis, 49 of 86 patients (57%) were well and alive. However, 35 patients died of OSCC carcinomatosis and two deaths were OSCC-unrelated. Therefore, only 84 patients' data were legitimate for survival analysis. The overall cumulative 1-, 3- and 5-year survival rates were 83, 66 and 56%, respectively.

### Expression of nm23-H1

**Figure 2:**
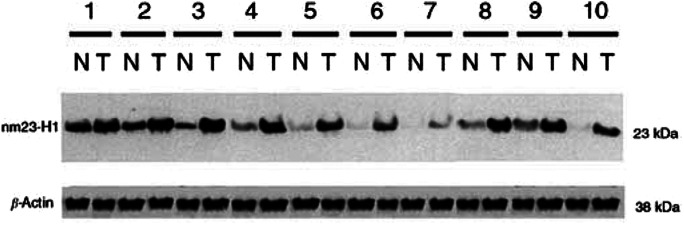
Immunoblotting analysis of nm23-H1 expression in the tumourous (T) and nontumourous (N) tissue pairs from 10 patients with OSCC. Extracts from tumour cells or normal oral mucosa derived from the same patients were electrophoresed on a 12% polyacrylamide gel with 4.5% stacking gel. After transfer onto a nitrocellulose membrane, the proteins were immunoblotted with 1 : 500 dilution of mouse monoclonal antibodies specific to nm23-H1, followed by HRP-conjugated goat anti-mouse IgG and development for enhanced chemiluminescence. Molecular weight (in kDa) is shown on the right.

Immunohistochemically, nm23-H1 was detected in 48.8%. (42 of 86) of tumourous specimens. IHC staining for nm23-Hl was predominantly cytoplasmic (Figure 1). To further confirm the nm23-Hl expression in OSCC, immunoblotting was carried out to compare 10 pairs of tumourous and nontumourous counterparts. The result was shown in Figure 2

and nm23-H1 was indicated as a 23-kDa band.

### Relationships between nm23-Hl expression and clinicopathologic variables

Relationships between nm23-Hl expression and seven respective clinicopathologic variables, for example, histologic grade of malignancy, tumour size, lymph node involvement, distant metastasis, tumour recurrence, habits of betel nut-chewing and cigarette-smoking, were summarised in Table 1

**Table 1:** Relationship between nm23-H1 expression and clinicopathologic parameters (*N*=86)

		**Expression of nm23-H1**		
**Clinicopathologic parameter**	**Number of patients**	**Negative (%)^a^**	**Positive (%)**	***P*^b^**
*Histologic grade of malignancy*				
Well-differentiated	50	25 (56.8)	25 (59.5)	0.972
Moderately differentiated	36	19 (43.2)	17 (40.5)	
				
*Tumour size*				
T1+T2 (⩽4 cm)	54	33 (75.0)	21 (50.0)	0.030
T3+T4 (>4 cm)	32	11 (25.0)	21 (50.0)	
				
*Lymph node involvement*				
Negative	43	17 (38.6)	26 (61.9)	0.052
Positive	43	27 (61.4)	16 (38.1)	
				
*Distant metastasis*				
Negative	73	34 (77.3)	39 (92.9)	0.086
Positive	13	10 (22.7)	3 (7.1)	
				
*Tumour recurrence*				
Negative	57	27 (61.4)	30 (71.4)	0.448
Positive	29	17 (38.6)	12 (28.6)	
				
*Betel nut-chewing habit*				
Negative	36	21 (47.7)	15 (35.7)	0.363
Positive	50	23 (52.3)	27 (64.3)	
				
*Cigarette-smoking habit*				
Negative	25	8 (18.2)	17 (40.5)	0.042
Positive	61	36 (81.8)	25 (59.5)	

a Percentage of patients in the category.

b *χ*^2^-test with Yates' correction.

. The nm23 expression correlated only with two clinicopathologic variables: tumour size and cigarette-smoking habit. In 65.6% (21 out of 32) of patients with larger-size primary tumours (>4 cm), while only in 38.9% (21out of 54) of those with smaller-size tumours (⩽4 cm) were nm23-Hl positive (*P*=0.03). Moreover, in patients with cigarette-smoking habit, nm23-Hl positive rate (68%, 17out of 25) was lower than those without this habit (41%, 25 out of 61) and the difference was significant (*P*=0.042). Although fewer patients with nm23-Hl-positive OSCCs had cervical lymph node involvement (37.2 *vs* 62.8%) or distant metastasis (23.1 *vs* 76.9%) than those with nm23-H1-negative tumours, these differences were marginal (*P*=0.052 and 0.086, respectively). Nonetheless, nm23-Hl expression did not correlate with histologic grade of malignancy, tumour recurrence or betel nut-chewing habit.

### Effect evaluation of nm23-Hl in 54 OSCC patients at the early T stage

When we analysed the data only from patients at the early T stages (tumour size ⩽4 cm), our results showed that the incidence of cervical lymph node involvement was indeed higher in the nm23-Hl negative group (51.5%, 17 out of 33) than the nm23-Hl positive group (9.5%, two out of 21) and the difference was statistically significant (*P*=0.004). Moreover, cigarette-smoking habit remained as a significant factor for suppressing nm23-Hl expression (*P*=0.022) (Table 2

**Table 2:** Relationship between nm23-H1 expression and clinicopathologic parameters of patients with the early T-stage OSCC (*N*=54)

		**nm23-H1**		
**Clinicopathologic parameter**	**Number of patients**	**Negative (%)^a^**	**Positive (%)**	***P*^b^**
*Histologic grade of malignancy*				
Well-differentiated	33	21 (63.6)	12 (57.1)	0.849
Moderately differentiated	21	12 (36.4)	9 (42.9)	
				
*Lymph node involvement*				
Negative	35	16 (48.5)	19 (90.5)	0.004
Positive	19	17 (51.5)	2 (9.5)	
				
*Distant metastasis*				
Negative	48	27 (81.2)	21 (100)	0.103
Positive	6	6 (18.2)	0 (0)	
				
*Tumour recurrence*				
Negative	41	22 (66.7)	19 (90.5)	0.095
Positive	13	11 (33.3)	2 (9.5)	
				
*Betel nut-chewing habit*				
Negative	29	18 (54.5)	11 (52.4)	1.000
Positive	25	15 (45.5)	10 (47.6)	
				
*Cigarette-smoking habit*				
Negative	15	5 (15.2)	10 (47.6)	0.022
Positive	39	28 (84.8)	11 (52.4)	

a Percentage of patients in the category.

b *χ*^2^-test with Yates' correction.

). Otherwise, no significant correlation was found between nm23-Hl expression and the other clinicopathologic factors, for example, histologic grade, tumour recurrence, distant metastasis and betel nut-chewing habit.

### Survival analysis of 84 OSCC patients

When all patients were divided into groups by each of clinicopathologic factors, significant difference by univariate analysis was shown in the following factors: tumour size (*P*<0.001), lymph node involvement (*P*<0.001), distant metastasis (*P*<0.001), and tumour recurrence (*P*<0.001) (Table 3

**Table 3:** Survival analysis of patients with OSCC (*N*=84)

	***P***	
**Clinicopathologic parameter (number of patient analysed)**	**Univariate analysis**	**Multivariate analysis**
*Degree of differentiation*		
Well-differentiated (49)	0.321	
Moderately (35)		
		
*Tumour size*		
T1+T2 (⩽4 cm) (53)	<0.001	0.002
T3+T4 (>4 cm) (31)		
		
*Lymph node involvement*		
Negative (42)	<0.001	0.747
Positive (42)		
		
*Distant metastasis*		
Negative (71)	<0.001	<0.001
Positive (13)		
		
*Tumour recurrence*		
Negative (55)	<0.001	0.040
Positive (29)		
		
*Betel nut-chewing habit*		
Negative (36)	0.173	
Positive (48)		
		
*Cigarette-smoking*		
Negative (25)	0.330	
Positive (59)		
		
*nm23-H1 expression*		
Negative (43)	0.150	
Positive (41)		

). In multivariate analysis, only tumour size (*P*=0.002), distant metastasis (*P*<0.001) and tumour recurrence (*P*=0.040) remained significant. No statistical difference was found in histologic grade of malignancy, habits of betel nut-chewing and cigarette-smoking, or nm23-H1 expression.

### Survival analysis of 53 OSCC patients at the early T stage

**Figure 3:**
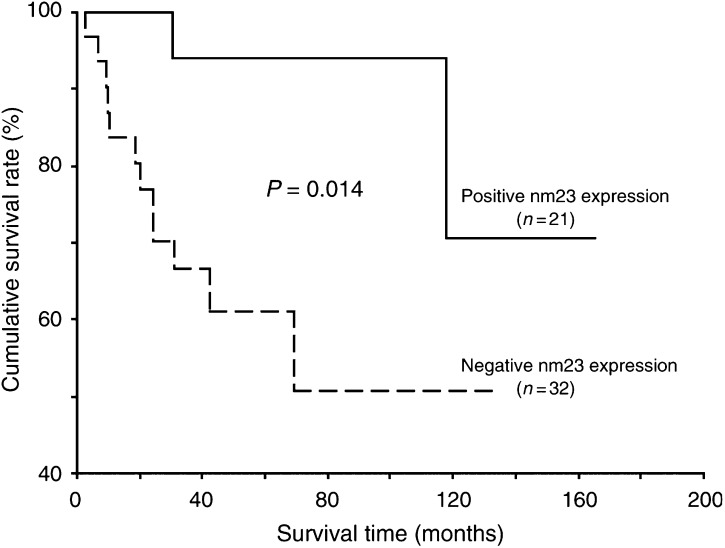
Overall survival curves of patients at the early T-stage OSCC in relation to tumour nm23-H1 expression. The survival analysis was assessed by Kaplan–Meier method and the difference in survival between positive and negative nm23-H1 expression groups was analysed by a log-rank test. The positive nm23-H1 group had significantly better survival than the negative nm23-H1 group (*P*=0.014).

When we analysed the data only from patients at the early T stages, statistical differences were found between survivals of two groups categorised by nm23-H1 expression (*P*=0.014) (Figure 3

) and cigarette-smoking habit (*P*=0.032), in addition to lymph node involvement (*P*=0.015), distant metastasis (*P*<0.001) and tumour recurrence (*P*=0.003). Nonetheless, in the multivariate analysis, only distant metastasis correlated with poor prognosis (*P*=0.013). The results are summarised in Table 4

**Table 4:** Survival analysis of patients with the early T-stage OSCC (*N*=53)

	***P***	
**Clinicopathologic parameter (number of patient analysed)**	**Univariate analysis**	**Multivariate analysis**
*Degree of differentiation*		
Well-differentiated (32)	0.443	
Moderately (21)		
		
*Lymph node involvement*		
Negative (34)	0.015	0.911
Positive (19)		
		
*Distant metastasis*		
Negative (47)	<0.001	0.013
Positive (6)		
		
*Tumour recurrence*		
Negative (40)	0.003	0.208
Positive (13)		
		
*Betel nut-chewing habit*		
Negative (29)	0.106	
Positive (21)		
		
*Cigarette-smoking*		
Negative (15)	0.032	0.165
Positive (38)		
		
*nm23-H1 expression*		
Negative (32)	0.014	0.230
Positive (21)		

.

## DISCUSSION

Our results demonstrated that decreased nm23 expression in OSCC patients might correlate with tumour metastasis, which was frequently associated with poor prognosis. These data suggested that nm23 could serve as a marker for disease progression and prognosis in OSCC patients.

In the present study, we found that the positive rate of nm23-H1 expression was significantly higher in OSCCs at the late T stage (T3, T4: tumour size >4 cm) than the early stage. Similarly, it has been indicated an association of nm23 overexpression with a larger tumour in neuroblastoma, thyroid, gastric and renal cell carcinomas (Hailat *et al*, 1991; Zou *et al*, 1993; Park *et al*, 1995; Müller *et al*, 1998). Increasing evidences have suggested that nm23 be involved in cell growth and microtubule mitotic spindle polymeration in S phase (Igawa *et al*, 1994; Caligo *et al*, 1995). Cipollini *et al*. (1997) also reported that downregulation of nm23 gene inhibited cell proliferation. However, not all of the above studies demonstrated a negative prognostic impact of nm23 overexpression or late T stage tumours (Zou *et al*, 1993; Müller *et al*, 1998). There were also some discrepancies among previous studies of the same tumours, such as gastric and colorectal cancers (Cohn *et al*, 1991; Haut *et al*, 1991; Kodera *et al*, 1994; Müller *et al*, 1998). Concerning gastric cancers, Kodera *et al* (1994) reported that a negative prognostic impact of decreased nm23 expression might correlated with increased lymphatic metastasis; while Müller *et al* (1998) showed nm23 overexpression was associated with aggressive tumour growth and poor survival. It is possible that nm23 could play variable roles in different molecular events and contribute to distinct outcomes. These results certainly need further investigations.

As noted previously, nm23, originally identified from a weakly metastatic cancer cells, was found to have antimetastatic potential in human carcinomas (Bevilacqua *et al*, 1989; Caligo *et al*, 1997). Since clinical prognosis of OSCC patients is closely associated with lymphatic spread and distant metastasis (Wang *et al*, 2002), it is reasonable to hypothesise that nm23 may play some role in disease progression of OSCC. Song *et al*, (2000) demonstrated that the increase of nm23 expression correlated with decreased incidence of lymph node metastasis in patients with head and neck cancers, though Pavelic *et al* (2000) revealed a different result in patients with the advanced disease. Such a variation may be due to heterogeneity of primary tumour distribution, methods of investigation and scoring systems for pathological variables. By showing that patients with larger OSCCs (>4 cm) mostly have nm23-H1 positive tumours, the current study indicated that nm23 might also be associated with cell proliferation (Caligo *et al*, 1995; Cipollini *et al*, 1997). Many studies have reported that late-stage cancer could obtain more metastatic ability by increasing events contributing to tumor spreading, such as angiogenesis, adhesion, proteolysis and motility (Petruzzelli *et al*, 1998; Bashyam, 2002). To avoid such interference, that increased size of primary tumour could escalate metastatic potential and then the data of patients at the late T stage might confound statistical analyses, we therefore particularly emphasised our analysis on patients at the early T stage. Our results indicated that nm23-H1-negative OSCCs indeed had a significantly higher incidence of lymph node metastasis at the early T stage. However, such correlation was equivocal at the late T stage (Table 1 and 2). These results suggested that the role of nm23-H1 in OSCCs at early T stage, including carcinogenesis, be different from that at late T stage, when the adjuvant therapy should be applied (Bevilacqua *et al*, 1989; Caligo *et al*, 1997; Cipollini *et al*, 1997; Pavelic *et al*, 2000; Song *et al*, 2000). As genetic instability altering with disease progression is one important character of malignant tumour, it is possible that nm23-H1 play various roles in the different stages of cancer development.

In fact, by an immunohistochemical study, Ohtsuki *et al* (1997) had demonstrated that OSCCs with positive nm23 expression was related with a lower incidence of lymph node metastasis. Lo Muzio *et al* (1999) further showed that this nm23 was nm23-H1. Our results supported their findings, in particular regarding patients at the early T stage; these data clearly indicated that nm23-H1 expression could have antimetastatic potential and prognostic significance in OSCC patients. Moreover, in addition to lymph node involvement, we evaluated the correlations of nm23 expression with occurrence of distant metastasis. Although statistical difference was not reached (*P*=0.086), a marginally higher metastatic incidence was observed in the nm23-H1 negative group (22.7%, 10 out of 44) compared with that in the nm23-H1 positive group (7.1%, three out of 42). As a matter of fact that the influence of nm23-H1 expression was clearly showed in the univariate analysis on OSCC patients at the early T stage, in which the survival of the nm23-H1 positive group was significantly better than that of the negative group. However, we could not found the similar result in patients at the advanced T stage. A larger study cohort is required to conclusively determine the effect of nm23-H1 expression on lymph node involvement, distant metastasis and cell proliferation, which may ultimately predict therapeutic response and survival.

In our results, it is worth noting that cigarette smoking might inhibit nm23-H1 expression and relate to poor survival for OSCC patients at the early T stage by univariate analysis. This effect on patients' prognosis was not obvious in the multivariate analysis. Our data indicated that smoking habit might be closely associated with cancer development and metastasis at the early T stage, but smoking alone was not a predicting factor for patients' response to adjuvant chemoradiotherapy at the late stage (Bundgaard *et al*, 1994). Furthermore, recent studies reported that mutagenicity of tobacco smoke might have some influence on biologic behaviors of OSCCs through DNA alterations, including K-*ras* oncogene and p53 mutation (Westra *et al*, 1993; Hsieh *et al*, 2001). The relationships between nm23-H1 expression and specific molecular targets of tobacco carcinogens need more investigation.

Recently, an elegant study by Iizuka *et al* (2000) supported a possibility by demonstrating that reduced nm23 expression could increase cisplatin resistance. These authors showed that downregulation of nm23-H1 expression could decrease intracellular cisplatin accumulation probably via altered Na^+^, K^+^-ATPase activity. If, as suggested by the above data, the nm23-H1-associated antimetastatic potential might be closely in company with chemosensitivity; it is possible that less nm23-H1 expression and hence higher cisplatin resistance may lead to poorer survival. Previous literature reported that metastatic tumors seemed more resistant to chemoradiotherapy (McKenna *et al*, 1990; Herbst and Langer, 2002; Real *et al*, 2002) and it might result from the common molecular mechanisms shared by both cancer metastasis and chemoradioresistance. The lack of nm23 expression in cancer cells might be another pathway leading to both events and has the potential of being a clinically prognostic predictor for chemoradiotherapy. In our series, local irradiation and/or systemic cisplatin-based chemotherapy were administered for the patients with metastatic cancers over cervical lymph nodes or distant organs. Referred to our database of OSCC patients, we will further study the role of nm23 in chemoradiosensitivity.

Our results revealed that there was no relationship between nm23-H1 expression and betel nut-chewing. A working group of the International Agency for Research on Cancer (IARC) concluded that evidence supporting a link between betel nut-chewing alone and human oral cancer was not sufficient (IARC, 1986). Buccal cancers were reported more frequently found in patients with betel nut-chewing habits (Thomas and MacLennan, 1992). However, tongue was only associated with cancers of smokers and the most common site among patients without any oral habits (Silverman and Griffith, 1972; Chen *et al*, 1999). In our series, there were very few OSCC patients with betel nut-chewing habit alone but without smoking (seven out of 86=8%) and most tumors were located on the tongue (58 out of 86=67%). Previous data reported that lingual cancers occurred less frequently in patients with betel nut-chewing habit alone (Ko *et al*, 1995; Chen *et al*, 1999). Furthermore, Taiwanese betel nuts do not contain tobacco and are different from those in India, where betel nuts nearly always include tobacco, a known cause of oral cancer. The above observations may explain the result that there was no significant relationship between nm23 H1 expression and the betel nut-chewing habit in our study.

In conclusion, our results showed that nm23-H1 was frequently expressed in pathologic specimens of OSCC. Nm23-H1 overexpression correlated with larger primary tumour size. In patients at the early T stage, increased nm23 expression was associated with decreased incidence of lymph node involvement, which in turn showed a better prognosis. This effect was suggested due to antimetastatic potential and the role in chemoradiosensitivity of nm23-H1. Nevertheless, other explanations are possible. The mechanisms of nm23-H1 about cancer metastasis and cytotoxicity of chemoradiation remain to be determined conclusively.
